# Novel therapeutic interventions towards improved management of septic arthritis

**DOI:** 10.1186/s12891-021-04383-6

**Published:** 2021-06-09

**Authors:** Jian Wang, Liucai Wang

**Affiliations:** 1 Department of Nursing, The Third Hospital of Jinan, Shandong Province, Jinan, 250132 China; 2 Hand and Foot Surgery, Shandong Provincial Hospital, Jinan, 250000 China

**Keywords:** Septic Arthritis, antimicrobial resistance, *Staphylococcus aureus*, Interventions

## Abstract

Septic arthritis (SA) represents a medical emergency that needs immediate diagnosis and urgent treatment. Despite aggressive treatment and rapid diagnosis of the causative agent, the mortality and lifelong disability, associated with septic arthritis remain high as close to 11%. Moreover, with the rise in drug resistance, the rates of failure of conventional antibiotic therapy have also increased. Among the etiological agents frequently isolated from cases of septic arthritis, *Staphylococcus aureus* emerges as a dominating pathogen, and to worsen, the rise in methicillin-resistant *S. aureus* (MRSA) isolates in bone and joint infections is worrisome. MRSA associated cases of septic arthritis exhibit higher mortality, longer hospital stay, and higher treatment failure with poorer clinical outcomes as compared to cases caused by the sensitive strain i.e methicillin-sensitive *S. aureus* (MSSA).

In addition to this, equal or even greater damage is imposed by the exacerbated immune response mounted by the patient’s body in a futile attempt to eradicate the bacteria. The antibiotic therapy may not be sufficient enough to control the progression of damage to the joint involved thus, adding to higher mortality and disability rates despite the prompt and timely start of treatment. This situation implies that efforts and focus towards studying/understanding new strategies for improved management of sepsis arthritis is prudent and worth exploring.

The review article aims to give a complete insight into the new therapeutic approaches studied by workers lately in this field. To the best of our knowledge studies highlighting the novel therapeutic strategies against septic arthritis are limited in the literature, although articles on pathogenic mechanism and choice of antibiotics for therapy, current treatment algorithms followed have been discussed by workers in the past. The present study presents and discusses the new alternative approaches, their mechanism of action, proof of concept, and work done so far towards their clinical success. This will surely help to enlighten the researchers with comprehensive knowledge of the new interventions that can be used as an adjunct therapy along with conventional treatment protocol for improved success rates.

## Background

Bacterial septic arthritis (SA) represents infection of the joints caused by the colonization of the joint cavity by a pathogenic bacteria. Acute bacterial arthritis represents an orthopedic emergency that needs early diagnosis and aggressive treatment to save the patient’s life and risk of irreparable joint degradation [1–4]. In some cases, septic arthritis may remain untreated and although the patient may survive the acute phase, but the chronic inflammatory condition may set in, which is altogether more challenging to treat [5–7].

Septic arthritis due to a pathogenic bacterial strain is mostly mono-articular involving one joint (as a typical red, swollen and painful joint). However, it may present itself as poly-articular as well (~22% of cases) [8–10]. Old age, diabetes mellitus, cirrhosis, renal disease, rheumatoid arthritis, osteomyelitis, prosthetic joint, recent joint surgery, concurrent skin infection, intravenous drug use are the possible risk factors [9, 11–13]. Looking at the incidence rates, SA exhibits an annual incidence of six to ten cases per 100,000 patients per year and an associated mortality rate as high as 10-15% [4, 14–19].

The possible entry points of bacterial inoculation in the joint include, three possible routes i.e. a) direct seeding due to recent arthroplasty surgery such as prosthetic implantation or fracture fixation or joint aspiration or intra-articular steroid injection, b) through the hematogenous route from a distant infection reservoir [20, 21] and c) due to extension of contiguous infection underlying bone infection in cases of osteomyelitis or prosthetic infected joint with involvement of biofilm bacteria [15, 22, 23]. Such biofilm bacteria exhibit a high degree of recalcitrance towards both immune attack and antibiotics, further complicating the clinical outcome [24]. Among the etiology associated with SA, *Staphylococcus aureus* has been the predominant pathogen responsible for 40%–50% of the cases of septic arthritis and further 6% to 22% of *S. aureus* isolates being identified as MRSA, hinting towards the frequent involvement of MRSA in bone and joint infections and its rise among orthopedic settings [25–29]. MRSA septic arthritis has been reported to be associated with longer hospital says, longer courses of antibiotic therapy, comparatively more number of surgical interventions [19, 30]. In fact, MRSA infection of joints were found more likely to be inadequately treated with ineffective and inappropriate empiric therapy as compared to MSSA infections adding to higher rates of treatment failures are seen [31, 32]. Another worrisome fact remains the increasing cases of community-acquired MRSA (CA-MRSA) mediated joint infections seen in young adults or children with no history of hospital stay or contact with healthcare staff [33–35]. CA-MRSA and strains display different genetic properties and different antibiotic patterns than hospital-acquired MRSA (HA-MRSA) and thus may require an altogether different set of prescribed antibiotics and treatment regimen [36, 37].

The second major challenge that calls for a search for improved management protocols is the destruction imposed by the exacerbated inflammatory process seen in patients with SA. This inflammatory response may turn chronic, causing persistent and irreversible damage to the affected joint and its architecture in a futile attempt to clear the bacterial infection [6, 9, 38–40].

With this scenario, the need to look into new alternatives for improved management of septic arthritis new adjunct therapies that can be given along with antibiotics represents a fruitful approach. Also, current research towards approaches for down-regulation of the heightened immune response to minimize the tissue damage is also required. The present review focuses to highlight the new treatment and management strategies which need further exploration to find clinical approval and success in the fight against SA. The major focus of the present review, however remains *S. aureus* which is the predominant problem pathogen and its recalcitrance emphasizes on the need to developing new strategies for effective management and treatment of *S. aureus* mediated arthritis**.**

## Methodology

PubMed as well as internet searches (Google search engine) were used without time restriction following use of related keywords and search terms such as MRSA, *methicillin-resistant Staphylococcus aureus*, *S. aureus,* biofilm, Septic arthritis, Phage therapy, Anti-microbial peptides applying combination of one or more key words in relevance to septic arthritis. For collecting data on proof of concept, more emphasis was laid on articles showing *in-vitro* and *in –vivo* efficacy studies (non-antibiotic interventions) related to bone and joint infections from 2010 onwards. Texts and authoritative Web sites were also reviewed. Articles were included if they were applicable to a) pathophysiology and disease course of septic arthritis b) management of septic arthritis c) phage therapy, AMP as antibacterial strategies and their mechanism of action d) non-antibiotic interventions used against bone and joint infections, e) anti-inflammatory management of bone and joint infections ie immunotherapies f) *in vitro*, *in –vivo* and clinical cases related to studying the potential of phage therapy, AMP’s, immunotherapies against septic arthritis and related to bone and joint infections (e.g osteomyelitis, prosthetic implant infections). Article pertaining to management of rheumatoid arthritis or osteoarthritis or non-bacterial causes of bone and joint damage were excluded.

Firstly, both the authors (JW, LW) screened the titles and abstracts of articles obtained from the initial search while excluding articles that did not fit into the context for this review. Further, full text of the shortlisted articles were read and relevant information abstraction performed. Information from the source documents was organized into various categories pertaining to and as per the sub-sections of the flow of the review article. Following information extraction, 189 citations were included in this review and the information was organized into the following relevant categories: Pathophysiology of Septic Arthritis, New intervention and management approaches that included AMPs, Phage therapy, Adjunct immunotherapies.

### Pathophysiology of septic arthritis : a closer look

The consensus for mainstay treatment for SA still remains the complete aspiration of purulent material followed by long-term (at least six weeks) of antibiotics, which begins as intravenous and then by oral administration [15, 41, 42]. This treatment regimen may help to tackle the emergency period, but the infection can persist, resulting in a chronic state that may gradually lead to permanent joint damage or degenerative joint disease [9, 43]. Therefore, before discussing the possible treatment options that are worth exploring, it is essential to understand the disease pathogenesis and what contributes to joint damage. The bacteria may gain entry into the joint space through either the hematogenous route or through direct invasion or possibly through the spread of a bone infection [41–44]. The synovial membrane has a complex architecture with dense vascularity but the vessels of the synovial intima do not have a limiting basement membrane. This allows passage of large molecules such as hyaluronic acid across the basement membrane essential to lubricate this articular cartilage [9, 13, 14, 45]. But, this also enables the constant contact with blood or lymph which facilitates hematogenous entry of bacteria or phagocytes carrying bacteria to enter the synovium. Once inside the synovial space, the bacteria adhere to the synovial cells and express host-derived extracellular matrix proteins (elastin, collagen, fibrinogen, fibrin, collagen, hyaluronic acid) that aid in bacterial adherence to joint tissue [23, 46, 47]. Also, certain bacteria, such as *S. aureus*, Streptococcus sp,. *Neisseria gonorrhoea* exhibit tissue tropism for the synovium [48]. Among the bacterial factors that mediate adherence is the “Microbial surface components recognizing adhesive matrix molecules (MSCRAMMs). *S. aureus* expresses a myriad of an adhesive surface protein termed as MSCRAMMS that play a vital role in adherence of the cocci to the joint matrix [49–51]. This MSCRAMM group includes Clumping Factor A and B i.e ClfA, ClfB; Sdr family of proteins (SdrC, D and E); Fibronectin binding protein (FnBPA, FnBPB), collagen adhesion (CAN), Bone sialoprotein binding protein (Bbp), Elastin binding protein (Ebp), autolysins A and E etc .[9, 50, 52, 53]. It was observed that mice which was infected with a mutant strain devoid for the collagen adhesin gene, showed 43% low occurrence of septic arthritis than in the corresponding wild type [54]. Moreover, past studies have depicted that collagen-binding protein (Can) was expressed by as high as 56% of *S. aureus* isolates associated with a bone infection showing its tropism for bone and collagen matrix [55]. In another study, mice vaccinated with a recombinant form of the adhesin showed significant reduction in the sepsis-associated mortality rate (13% vs 87% in non-vaccinated group) [56]. Similarly, guniea pigs infected with mutant *S. aureus* strain i.e defective in expression of fibronectin-binding protein showed three times less adherence to miniplates implanted in than normal wild type strains [57] stressing on the critical role that fibronectin-binding proteins (FbpA and FbpB) play in pathological course of SA. Once adhered, the bacteria multiply using the synovial milieu as an ideal culture medium. Recent evidence have suggested the existence of biofilm-like clumps or agglomerates for *S. aureus* and MRSA strains in the synovial fluid of patient suffering from chronic joint infections and septic arthritis [57–59]. Pestrak et al. [60] highlighted on the role of host factors such as fibrinogen and fibronectin in the formation of such biofilm-like aggregates within the joint fluid. These biofilm bacteria exhibit altered phenotypes called small colony variants (SCVs) that exhibit slow growth and such forms are capable of intracellular persistence within the osteoblasts, fibroblasts, neutrophils etc. This property enables the pathogen to evade the immune attack while displaying higher recalcitrance towards deployed antibiotics [61–64]. Such biofilm clumps in the synovial fluid and nearby tissue act as communicating niches of hiding bacteria that may later re-populate to new sites leading to a second wave of re-infection and re-seeding [65, 66]. Besides this, *S. aureus* secretes virulence factors such as enterotoxins, protein A, capsular polysaccharide (aid in evasion from phagocytosis and promote intracellular survival of capsular strains) along with staphylococcal toxic shock syndrome toxin (TSST-1) that acts as superantigen for non-specific activation of a large number of T-cells play important role in progression of the disease [67, 68]. It has been studied that α-hemolysin also causes blood coagulation, platelet aggregation, neutrophil adherence and lymphocyte DNA degradation [69]. CA-MRSA expresses Panton-Valentine toxin (unlike HA-MRSA) which enables the cocci to survive within the neutrophils and even multiply, thus contributing to the development of fulminant joint infection even in young healthy children and adults [70, 71]. It is associated with MRSA cases that cause more invasive osteoarticular disease requiring higher surgical interventions, longer stay in hospital and increased rates of septic shock and prolonged antibiotic treatment [72].

**Fig. 1:**
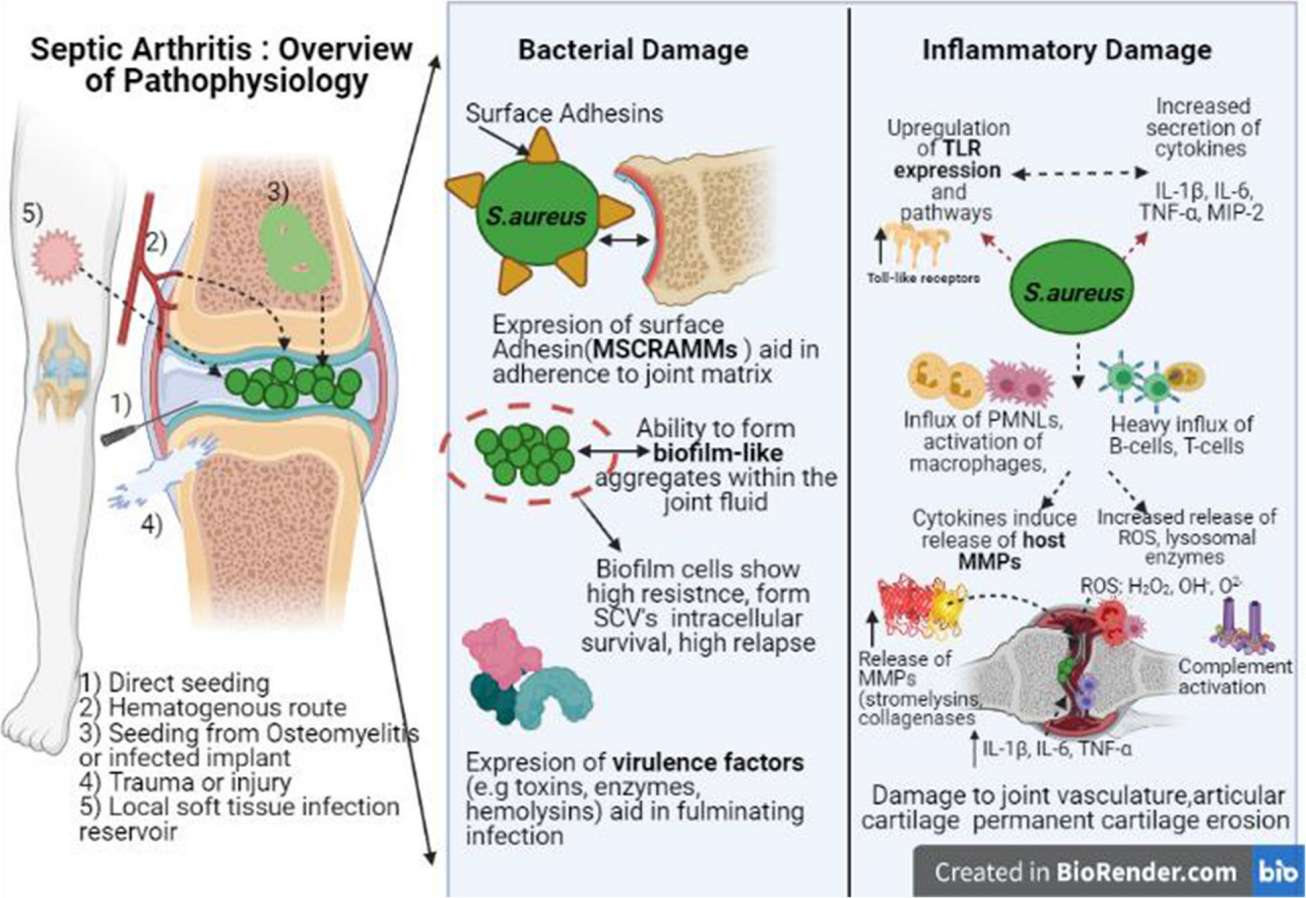
Schematic illustration of the pathophysiology and damage involved in septic arthritis. (MSCRAMMs; microbial surface components recognizing adhesive matrix molecules, MMPs; Matrix metalloproteinases, ROS; reactive oxygen species, TLR: Tolle like receptors)

Following a bacterial seeding, the bacterial products and toxins initiate the inflammatory cascade characterized by an influx of immune cells, neutrophils , activation of macrophages, and release of inflammatory cytokines such as IL-1β, IL-6, TNF-α, MIP-2, Granulocyte-macrophage colony-stimulating factor (GM-CSF) [9, 43]. Toll-like receptors (TLR’s) play a key role as transmembrane proteins involved in recognizing bacterial pathogen-associated molecular pattern molecules (PAMP’s) and up-regulated expression of TLR’s results in nuclear translocation and activation of transcription factor NF-kB that further promotes secretion of pro-inflammatory cytokines and neutrophil infiltration [73–75]. These cytokines recruit more phagocytic cells to the site and also activate host C-reactive protein (CRP) levels of the liver which in turn activate the complementary pathways. The phagocytosis of bacteria by macrophages, synoviocytes, PMNL’s release more lysosomal enzymes and reactive oxygen species (ROS) and further induction of cytokines, leading to the development of redness, swelling, pain [23, 39, 76]. This inflammatory reaction mounted by the host is protective and along with antibiotic therapy may help to contain the spread of infection further. But, if in case the infection is not cleared, there continues an ongoing battle of the host mounted an immune response against bacteria and the immune system exacerbates rather than ameliorates the outcome of septic arthritis [2, 13, 21, 77]. Soon, the T-cells also start to enter the joint cavity and get activated upon antigen presentation supported by host antigen-presenting cells (APC’s). The heavy influx of T-cells, B-cells, macrophages causes thickening of the synovial membrane. High levels of cytokines induce the release of host matrix metalloproteinases (stromelysin and collagenases) and lysosomal enzymes, which further worsen joint degradation [78, 79]. As the intra-articular pressure rises, the synovial vasculature may get compressed with thrombosis and further permanent damage to the articular cartilage impeding the blood and oxygen supply as well. This may extend to the articulating bone resulting in serious damage to bone growth, especially in children, and causing permanent cartilage erosion [13, 43, 80]. A schematic illustration of the pathophysiology and damage involved is presented in Fig.1.

### New intervention and management approaches

The new approaches against SA have been divided broadly as anti-bacterial strategies’ and immune based management options that are worth exploring. Each approach has been discussed in terms of its mode of action and supported with recent data (*in vitro* and *in vivo* studies) and discussion of the major findings.

### Anti-microbial peptides (AMP’s)

Antimicrobial peptides also referred as host defense peptides (HDP’s) present a promising class of anti-bacterial agents that exhibit potent antimicrobial activity against broad range of pathogenic microorganisms. AMPs are highly conserved, short (15-50) amino acid sequences that are effector molecules of the innate immunity and can be of plant, animal or microbial origin [81, 82]. AMP’s are mostly cationic peptides that show rapid killing and are effective against range of drug-resistant pathogens [83, 84].

#### Mechanisms

**Fig. 2:**
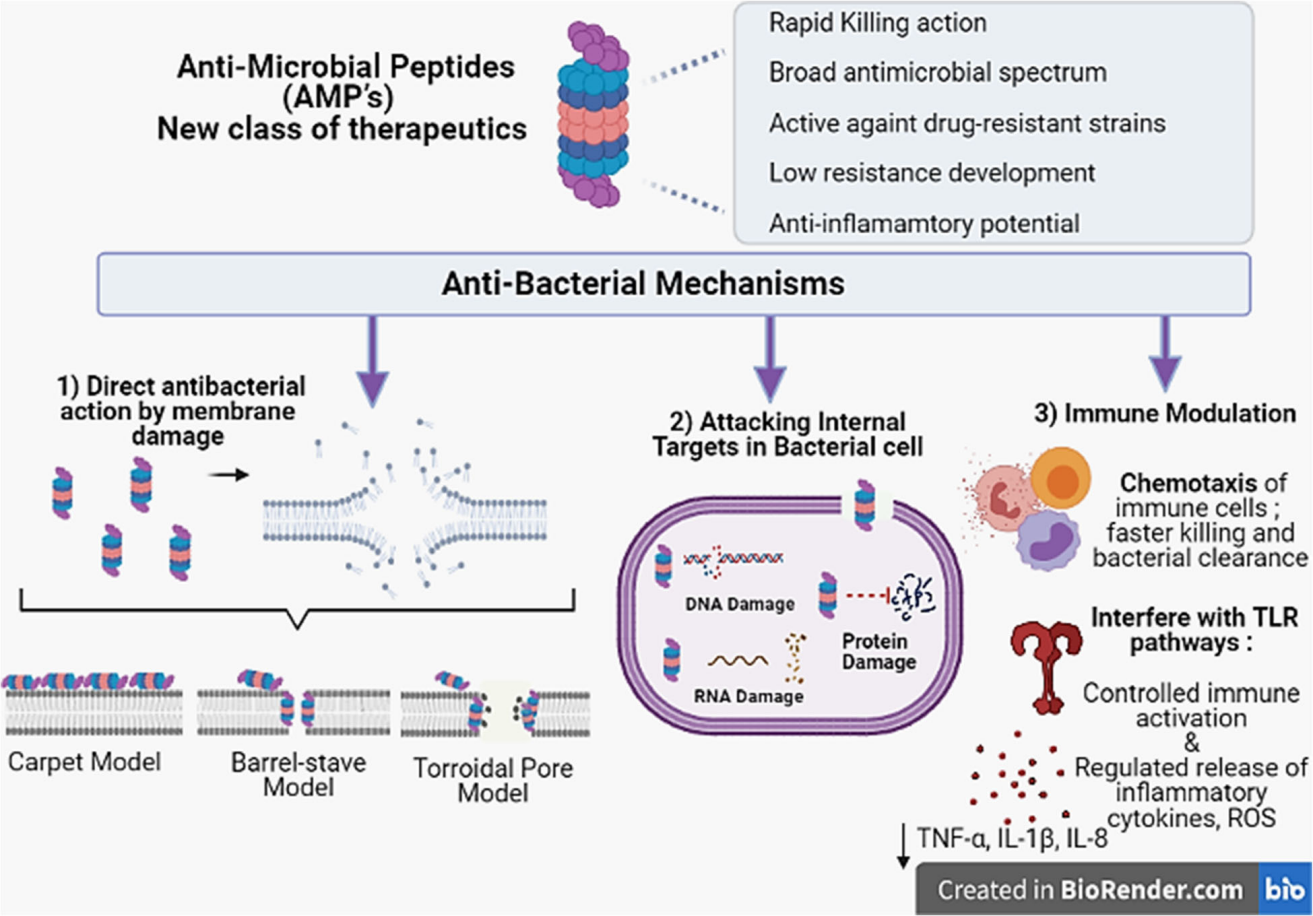
iagrammatic illustration of the membrane and non-membrane mechanisms adopted by AMP’s to kill bacteria. (TLR: Toll-like receptors, ROS: Reactive oxygen species, TNF: Tumour necrosis factor, IL: Interleukin)

The classical mode of action of these peptides includes direct killing via both membrane targeting as well as non-membrane targeting routes (Fig.2). The membrane targeting includes binding of the AMP through its binding domain to the bacterial membranes. The outer surface of both Gram-positive and Gram-negative bacteria contain structures [Lipopolysaccharide (LPS), phospholipids, teichoic acids etc.] that impart a net negative charge allowing easy electrostatic interaction with cationic AMP’s. Once bound, the next step includes the formation of membrane-embedded pore within the lipid bilayer (as explained through torroidal, barrel stave or carpet model) [85–87]. Few AMP’s have also been shown to cause damage by targeting internal targets within the bacterial cell such as disruption of vital proteins, enzymes, DNA/RNA, protein folding etc. [88–90] without causing substantial membrane permeabilization but still leading to cell death.

Besides, the membrane damage and direct killing, the other mechanisms recently highlighted include the immune-modulatory ability of AMP. The immunomodulatory functions displayed by AMPs include: chemotaxis of immune cells thus aiding faster clearance and more effective resolution of resident bacteria, activation of immune cells but in a regulated and controlled manner, interference with Toll-like receptor (TLR) pathways that mediate the release of pro-inflammatory cytokines and reactive oxygen species, induction of anti-inflammatory cytokines essential to optimize the heightened inflammatory process, scavenging of bacterial endotoxins and their inactivation as well as promoting wound healing and angiogenesis etc. [91–93]. The major class of AMP i.e cathelicidins and defensins act as potent chemoattractants via their ability to bind chemokine receptors, leading to activation and recruitment of several immune cell types, including monocytes, neutrophils, dendritic cells and also the T-cells [94–96]. This recruitment may actually enhance the bacterial clearance. Also, the immune cells recruited by AMPs may also, play a regulatory function in balancing the inflammatory process. Secondly, AMPs modulate the activation of macrophages and dendritic cells by interacting with specific Toll-like receptor (TLR) ligands and by perturbing their pathways thus preventing undue activation of macrophages and dendritic cells [97–99]. For example, LL-37 suppresses the TLR-2 and TLR-4 induced production of TNF-α, IL-1β, IL-8 [97, 100]. Similarly, the 13-mer Indolicidin, inhibited *Escherichia coli* O111:B4 LPS **i**nduced TNF- α secretion even when added at a delay of one hour [101]. To add to this, innate defense regulators (IDR) which are bactenecin-derived host defense peptides such as IDR-1002 have also shown to down-regulate the heightened immune response by decreasing production of TNF-α, IL-6, IL-8, and nitric oxide which is triggered by TLR ligands [102, 103]. Another synthetic peptide i.e IDR-1018 and clavanin A increased the levels of anti-inflammatory cytokine IL-10. In addition, AMP’s are able to scavenge the endotoxin LPS thus preventing the binding to TLR4 and further inflammatory activation [104]. LPS-scavenging and anti-endotoxin activity was reported while studying frog dermaseptin-derived peptides [105] and thrombin-derived C-terminal peptides (TCPs)].

AMP’s can thus work both as anti-bacterial killing peptides as well as participating in normalization of the deranged immune that causes significant tissue damage (even after acute episode has subsided) and thus represent an ideal option in the fight against management and treatment of SA. Moreover, AMP’s have been shown to work in a synergistic fashion when used together with conventional antibiotics [106, 107] making them ideal to be used as an adjunct therapy.

### AMP’s and Septic Arthritis

Although the research targeting use of AMP’s against bacterial arthritis is scarce, we present few important findings. Varoga et al .[108] reported enhanced expression of human β-defensin-2 (HBD-2) in synovial membranes that were exposed to bacteria (using a stable synoviocyte line K4IM) mimicking a case of septic arthritis. The study showed that bacteria-colonized synovial membranes displayed comparatively higher levels of human β-defensin-2(HBD-2) peptide than unexposed samples. This suggests the involvement of these cationic peptides in intra-articular defense mechanisms and their role in regulating the pathological course of septic arthritis.

Studies also highlighted the protective role of AMP’s (HBD1, HBD2) as part of innate system of the articular cartilage against infection and emphasized that purified or recombinant AMP’s represent potential therapeutic agents that can be administered in septic arthritis to further boost the innate resistance system of the synovial tissue [108–110].

Recently, Elizagic et al. [111] investigated the antimicrobial activity of peptides derived from C-type Lectin Domain Family 3 Member A (CLEC3A) against septic arthritis. CLEC3A is a cartilage-specific protein that is present in articular cartilage and also in growth-plate cartilage tissue in both resting and proliferating types. Researchers designed peptides and recombinantly expressed CLEC3A domains and in vitro assays using these recombinant peptides exhibited significant killing activity against *E. coli*, *P. aeruginosa* and *S. aureus* by membrane pore formation and permeabilization and also showed reduced bacterial adhesion when titanium implants were coated with recombinant CLEC3A peptides. This hints towards their potential therapeutic use against prosthetic-induced cases of septic arthritis. Another advantage offered is that since CLEC3A-derived peptides are normally expressed in the articular cartilage system under physiological conditions, using them would lead to no or minimal immunogenic reaction thus prolonging their retention *in vivo*.

Another study by Ries and co-workers [112] studied anti-bacterial efficacy of LyeTxI-b, a synthetic peptide derived from *Lycosa erythrognatha* spider venom in mice model of *S. aureus*-induced arthritis. Results indicated that Lye Txl-b was able to significantly reduce the bacterial load in the affected joint space and the simultaneous reduction in the number of inflammatory cell recruitment in the bacteria challenged joint. Also, when co-therapy of Lye TxI-b was given with clindamycin, a higher reduction in the levels of IL-1β cytokine (a major cause of tissue destruction) and CXCL1 chemokine in the joint were observed when compared to non-treated joints. This indicates towards potential role of this peptide as a promising adjunct strategy for better control of infection and inflammation in SA. Table 1 delineates the recent new developments pertaining to improved delivery and efficacy of various AMP’s showing potency against the pathogens commonly associated with bone and joint infections.

**Table 1:** New Developments related to AMP research against Bone and Joint Infections

AMP	Study Highlights	References
Engineered chimeric bifunctional peptides (TiBP1-GGG-AMP, and TiBP2-GGG-AMP)	• Two bi-functional chimeric peptides synthesized with an aim to strongly bind to titanium substrate (high affinity) while retaining antimicrobial motif free. • Significant reduction in bacterial adhesion and colonization of *S. aureus*, *S. mutans* and *E. coli* as tested.	[113]
OP-145 incorporated into Polymer-Lipid Encapsulation Matrix (PLEX)-coating	• Breij and co-workers incorporated a synthetic peptide OP-145 into PLEX coating to obtain high peptide levels for prolonged periods at the implant-tissue interphase. • PLEX coated nails inserted into rabbits inoculated with *S. aureus*. • Result showed sustained release of OP-145 from plex coatings into the joint space and around the nails. • Effective resolution of induced infection in 67% of test animals within 28 days as shown by culture tests.	[114]
Five Artificial Peptides synthesized from optimized peptide library	• Bormann and co-researchers synthesized short artificial AMPs using solid phase peptide library. • Later, studied their anti- biofilm potency and their effect on human osteoblast cells. • Peptides showed marked reduction in biofilm formation by *S. aureus*, *E.coli*, *P. aeruginosa*, MRSA and MSSA strains as tested by microcalorimetry and FISH. • Peptides able to significantly reduce internalization of bacteria within osteoblast cells with no effect of viability of human osteoblast cells.	[115]
Novel in house designed potent ultrashort AMP i.e RBRBR	• Research team developed novel in house designed potent ultrashort AMP i.e RBRBR and encapsulated it in chitosan based nanoparticles using ionotrophic gelation method (RBRBR-CS-NP). • Encapsulated peptide showed progressive sustained release till 14 days. • Signficant decrease in *S. aureus* counts by three log counts with 98% inhibition of biofilm formation. • No toxicity against mammalian cells and human erythrocytes.	[116]
LL-37	• Kang and co-researchers developed 24 hour S. aureus biofilm on cobalt chromium discs followed by treatment with LL-37, AgNP’s and conventional antibiotics combinations. • LL-37 effective in decreasing counts of S. aureus by as high as four log reduction in CFU and this was even more than combination groups i.e AgNP’s and rifampin and even combination of gentamycin and rifampin. • Potential application of LL-37 against bone and joint related biofilm mediated infections strongly advocated.	[117]
HHC36 peptides	• Chen and co-workers developed a Pandora box approach i.e a novel system promoting on demand release of AMP in and around the affected joint area and implant when bacterial infection occurs and lowers the surrounding pH. • This Pandora box was loaded with HHC36 peptide inside the specially designed titania nanotubes (Ti-NTs) nanotubes and “closed” (surface-modified) with a pH-responsive molecular gate. • The poly (methacrylic acid) (PMAA) swelled under normal conditions (pH 7.4) and collapsed under bacterial infection when pH drops below 6.0 allowing release of AMPs to kill bacteria immediately. • This approach exhibited excellent activity against MRSA, *E. coli*, *P. aeruginosa* thus representing a novel smart drug delivery technology worth exploring.	[118]
Romo1-Derived Antimicrobial Peptide (AMPR-11)	• Lee and team developed AMPR-11, the antimicrobial peptide (AMP) derived from mitochondrial nonselective channel Romo1. • Peptide represents a novel class of fast acting AMP exhibiting broad spectrum antibacterial activity against range of clinical pathogens and multi-drug resistance (MDR) strains. • Exhibits unique mechanism of killing which includes bacterial membranes by interacting with cardiolipin and lipid A. • Exhibited significant activity against intracellular invading bacteria and superior *in vivo* efficacy in murine model of sepsis.	[119]

### Phage therapy: a new era of treatment

The use of lytic bacteriophages to kill pathogenic bacteria is referred to as phage therapy. Bacteriophages perfectly fit into the class of safe and potent antimicrobial agents and the reasons are many-fold. Firstly, from a clinical standpoint, phage therapy represents a safe approach and exhibits little or no history of adverse effects or tissue toxicity [120, 121]. Secondly, phages owing to their self-replicating nature exhibit the unique property of auto-dosing at the expense of host-pathogen [122, 123]. Thirdly, phages being selective in their action, do not alter or disrupt the normal flora, unlike long-term antibiotic-based therapy which poses significant damage to the body’s normal flora. Phages also exhibit synergy when given along with antibiotics as co-therapy, further decreasing the frequency of emergence of resistant mutants [124–126]. Phage-based therapeutics have re-surfaced again as they exhibit potent efficacy against a range of bacterial infections especially those caused by drug-resistant strains and this approach warrants further work [127–129].

#### Mechanisms

Lytic phages work as killing machines. They start their process of infection after adsorption to their particular host bacterium through specific receptors. Soon after attachment, they inject their genome into the host cytoplasm and utilize the host’s proteins and machinery to reproducing within and assemble into a large number of progeny phages. Finally, the new progeny phages lyse their host bacteria and get released to start another round of infection. Besides this conventional mode of killing, phages also exhibit anti-biofilm activity [130, 131]. This is particularly important as recent studies have highlighted on the presence of free-floating bacteria in clumps or as biofilm-like aggregates seen in the synovial fluid of infected joints and being involved with the pathogenesis of infectious arthritis [24, 57, 132] Dastghyeb et al .[58] demonstrated that methicillin resistant *S. aureus* (MRSA) was able to form biofilm-like aggregates seen in human synovial fluid (SF) present within the joint cavity. These biofilm-like agglomerations tend to decrease the ability of the neutrophil-mediated killing of the cocci within the synovial fluid [57] while making the pathogen more recalcitrant. Phages are equipped with virion-associated de-polymerases and peptidoglycan degrading endolysins that degrade the biofilm matrix, penetrating the deeper layers of biofilm and attacking bacterial cell walls [132, 133]. Phages are also able to bind and lyse the bacteria that are metabolically dormant or the slow-growing persister cells (low-metabolic cells) that may reside within the deeper layers of host tissue [134]. These slow-growing cells exhibit altered phenotypes and often escape from the attack of antibiotics and thus re-emerge to start another round of infection days or months after the primary antibiotic therapy has stopped. *S. aureus* and *S. epidermidis* are known to form small colony variants (SCVs) that have been found associated with fibroblasts in joint infections and these SCV’s are responsible for intracellular persistence, re-infection, and treatment failures [63, 64, 135]. Evidence studies report that *S. aureus* is also able to survive intracellular and successful internalization within the bone cells, fibroblasts, osteoblasts, macrophages, epithelial cells [136–140] and by this, it may evade its clearance from the immune cells as well as from the administered drugs. However, studies have indicated towards the ability of phages to penetrate the eukaryotic cells and attack the intracellular populations of pathogens that may hide within, thus reducing recurrent infections [126, 141].

**Fig. 3:**
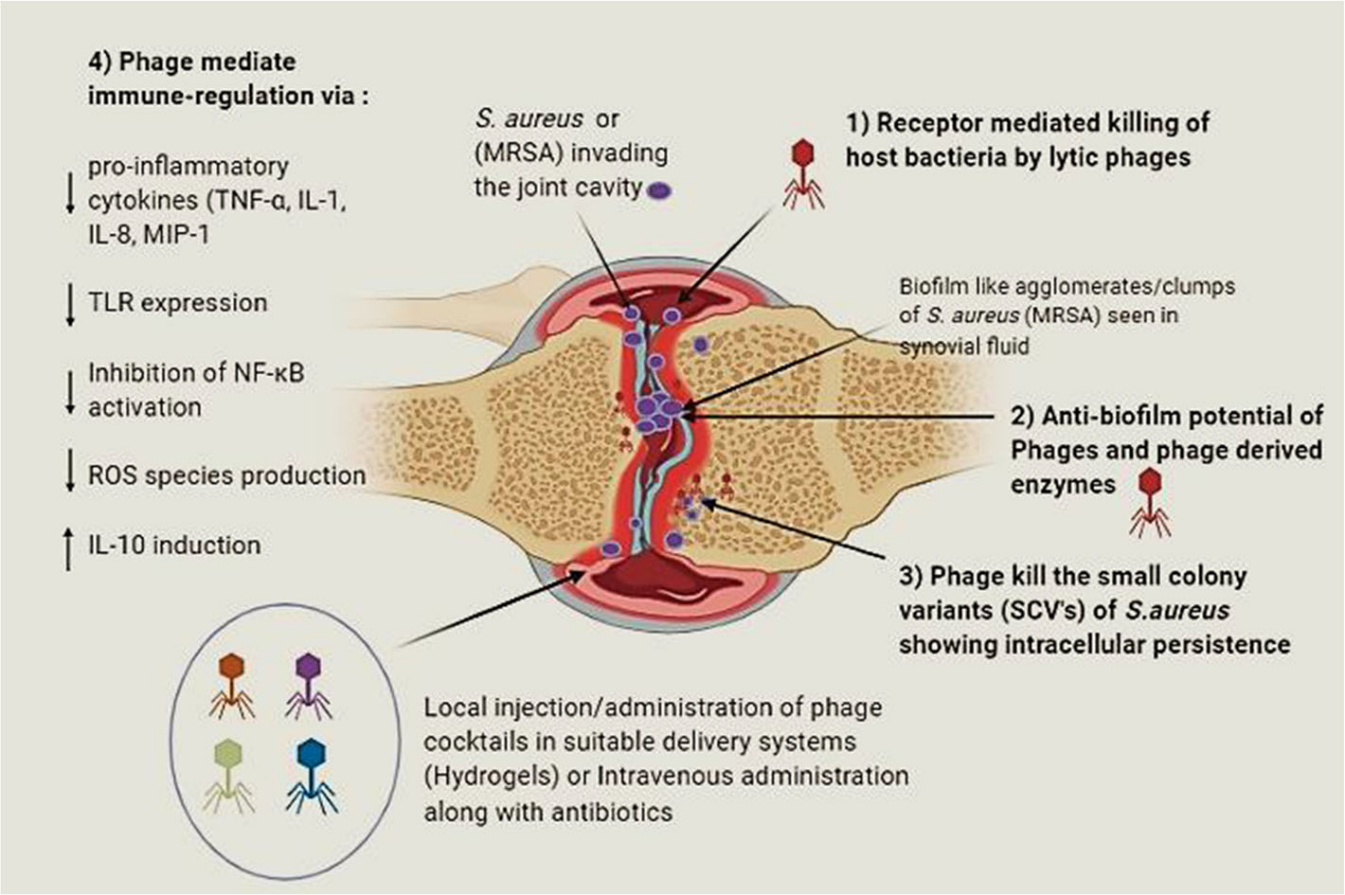
Detailed representation of the different mechanisms through which phage may help to resolve septic arthritis infection. Image created using Biorender (SCV’s: small colony variants, TNF: tumor necrosis factor, TLR: toll-like receptors, NF-κB : IL-Interleukin, ROS: reactive oxygen species; MIP: macrophage Inflammatory Proteins)

Another major aspect of phages is their potential role (other than lytic) in modulating the immune system at different levels. Studies report that phage therapy can help to correct the heightened levels of inflammation seen in many infections in different ways [142–146]. These may include; 1) the ability of phages to reduce high levels of pro-inflammatory cytokines (TNF-α, IL-1, IL-8, MIP-1), 2) through LPS binding 3) inhibition of excessive reactive oxygen species (ROS) species production and 4) induction of synthesis of potent anti-inflammatory cytokine essential in limiting cell and tissue injury during bacterial infections IL-10. Phage ISP specific for *S. aureus* phage showed induction of anti-inflammatory IL-1 receptor antagonist (IL-1RA) synthesis by human monocytes thus leading to the repression of pro-inflammatory cytokines [147]. Phages have also been shown to down-regulate the expression of TLR4 and TLR2 expression which are key sensors involved in the detection of *S. aureus* pathogen via interacting with specific PAMPs that leads to activation of NF-κB, leading to cytokine production, cell infiltration, phagocytosis etc .[148, 149]. Zhang and co-workers [150], *S. aureus* phage vB_SauM_JS25 inhibited the production of pro-inflammatory cytokines possibly via inhibiting the NF-κB (a key transcription factor for encoding pro-inflammatory cytokines) phosphorylation which led to the decline in the levels of inflammatory cascade mediators. Thus, it is evident that besides its killing ability, phages also lead to down-regulation of excessive immune responses, thus contributing to the maintenance of immune homeostasis. A detailed representation of the different mechanisms through which phage may help to resolve septic arthritis infection have been depicted in Fig.3**.**

### Phage therapy and SA

Phages have been tested against bone and joint infections including osteomyelitis and prosthetic or orthopedic implant infections but studies strictly focused on the use of phages in resolving cases of acute septic arthritis is still scarce. However, conditions such as osteomyelitis or bacterial colonization of orthopedic implants closely mimic and act as important predisposing conditions that may lead to septic arthritis involving the joint space. Table 2 depicts the outline of recent *in-vitro, in-vivo* and clinical cases wherein phages showed excellent efficacy in treating various bacterial infections of bone and joints (including those caused by MRSA and other drug-resistant strains).

**Table 2:** Recent studies demonstrating the therapeutic success of phage therapy in treatment of bone and joint infections

Outline of Study	Major Findings	Reference
Therapeutic efficacy of cocktail of five phages against *S. aureus* given intraperitoneally at a dose of 10^8^ PFU/ml in a rat model of joint infection (alone as well as along with vancomycin)	• Phage treatment alone led to 5-fold reduction in bacterial load in the peri-implant tissue. • When given in combination with vancomycin, 6.2 fold reduction occurred. • 22.5 fold decrease in bacterial burden in the joint tissue unlike sham treated animals. • Phage treated animals showed marked reduction in swelling and joint inflammation.	[151]
• Therapeutic efficacy of MRSA phage MR-5 given single as well as co-therapy with linezolid in resolving MRSA mediated implant infection in mouse model of post arthroplasty joint infection. • Phage MR-5 alone as well as mixed with linezolid was encapsulated in biodegradable HPMC gel and coated onto K-wires. • Coated and uncoated K-wires inserted into the mice femur followed by MRSA inoculation.	• Dual Hydrogel based system exhibited release of both agents i.e phage and linezolid in a slow sustained manner in the joint tissue • Combination therapy showed synergistic effects. • Highest decrease in bacterial burden, improvement in joint mobility and lowered cytokine levels seen in combination group.	[126]
• To evaluate efficacy of *S. aureus* specific bacteriophage cocktail formulations against MRSA employing rabbit model of osteomyelitis. • Phage therapy initiated 3-6 weeks post development of experimental osteomyelitis. • Test animals received four repeated doses of seven MRSA phages in a cocktail mix given at the interval of 48 h.	• Test rabbits recovered from the infection within two weeks with marked decline in local oedema, erythema and induration. • Phage treated group showed new bone formation and improved histopathology.	[152]
• **Clinical case study** of salvage phage therapy in a 72 year old male with a chronic MRSA prosthetic joint infection. • Infection persisted even after two DAIR procedures. • Patient administered three doses of 2.7 × 10^9^ PFU through *i.v* route along with daptomycin.	• Phages were able to sterilize the patient’s severe chronic MRSA joint infection with a single virulent bacteriophage given i.v for three days giving negative cultures.	[153]
**Clinical case:** • 42-year old man with multidrug-resistant left tibial infection was positive for multidrug resistant strains of *Klebsiella pneumoniae* and *Acinetobacter baumannii.* • Patient received combination therapy of bacteriophage (intravenous bacteriophage therapy at 10^7^ PFU/mL titers) and antibiotics.	• Within days of phage administration, the patient showed improved wound healing, decrease in the chronic bone pain. • Negative bacterial cultures obtained for both the causative bacteria and patients’ leg was thus saved from amputation surgery.	[154]

Septic arthritis represents an orthopedic emergency which is tackled by surgical interventions along with high doses of intravenous antibiotics given immediately within the first 24 hours [7, 155, 156]. Phages can also be administrated intravenously during this emergency period in order to provide more effective control of the infection process and associated sepsis as demonstrated in past studies [122, 157–160]. In a recent study by Ferry and co-workers [161], a cocktail of *S. aureus* specific bacteriophages (10_10_ PFU/ml) impregnated in hydrogel were given to a 49-year old patient suffering from mega-prosthetic infection as part of salvage therapy along with debridement, antibiotics and implant retention (DAIR). The selected phages showed sustained release from the hydrogel with stable titers for at least 6 h showing a significant reduction in numbers *in vitro*. This approach clearly demonstrates the feasibility of the use of bacteriophage cocktails along with surgical interventions that can be administered either intravenously along with antibiotics or can even be placed locally entrapped within such hydrogel systems during the debridement procedure adopted. A similar approach was used with PhageBioderm, a polymeric bandage that was directly placed at wound site allowing the release of phages slowly over an extended period aiding in healing of infected venous ulcers and poorly healing wounds [162, 163]. Another possibility can also be a direct injection of phage preparations into the affected joint lesion area for improving phage targeting inside the body and also reducing the issues related to systemic phage clearance from the blood [152]. Phages have been successful in saving patient’s life from overwhelming sepsis as reported by recent studies. Dupplesis et al. [164] reported the successful administration of phage cocktail against refractory *P. aeruginosa* bacteremia in a 2-year-old boy that showed allergy to antibiotics. Phage cocktail was given intravenously every 6 h for a period of36 h. Blood cultures turned and thus phage cocktails were able to bring complete sterilization of the blood. In another case of acute *P. aeruginosa* septicemia, 50 ml of lytic phage cocktails were administered in a 6 h *i.v* infusion for 10 days. Post-phage therapy, the blood cultures turned negative, and a drop in CRP with disappearance in fever were seen within days [165]. These findings indicate the possible role of phage administration in cases of septic arthritis both during and after the emergency period to prevent joint damage, acute sepsis, and mortality and development of chronic SA.

### Adjunctive immunotherapies

**Fig. 4:**
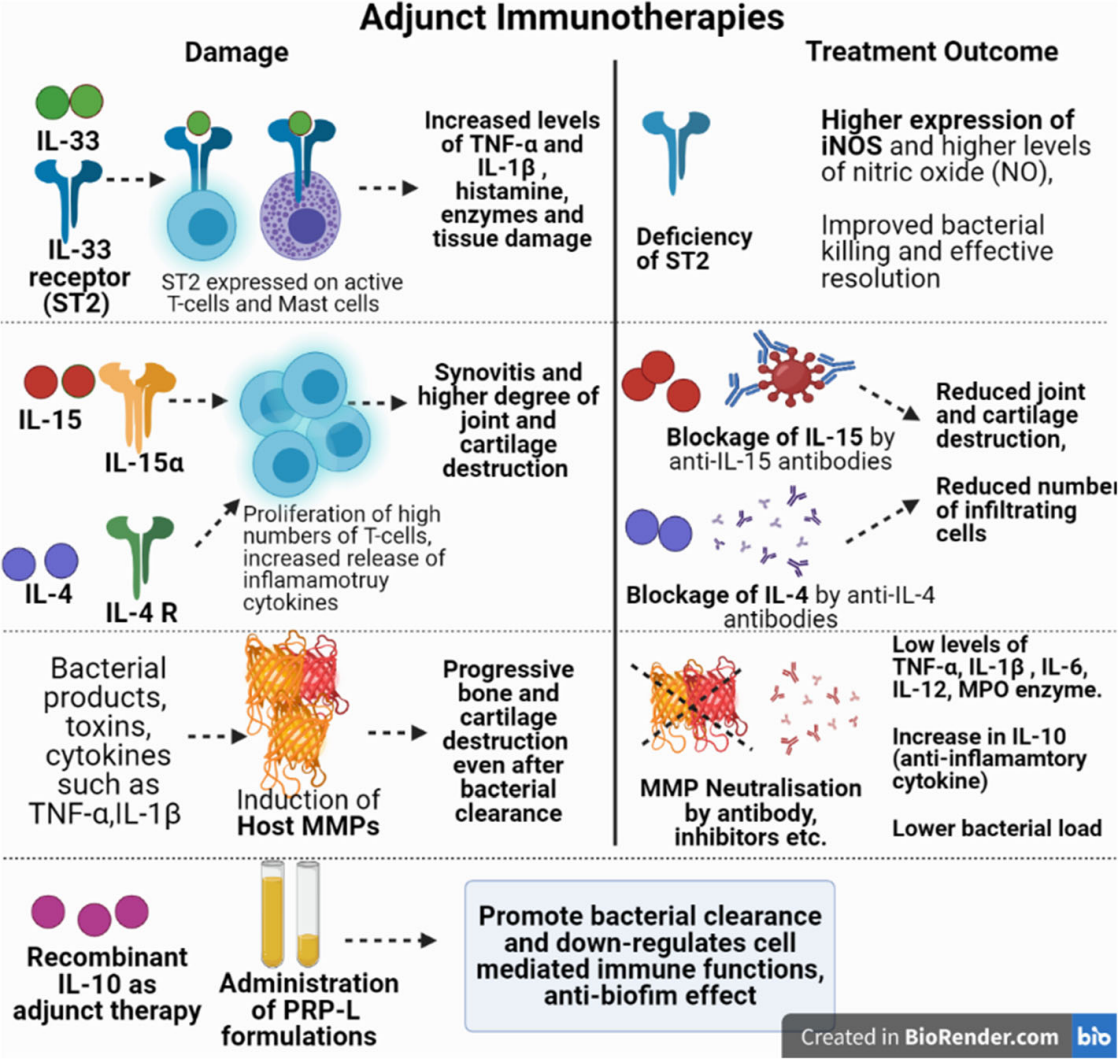
Schematic illustration of the various adjunct immunotherapy based treatments against septic arthritis (iNOS: Inducible nitric oxide synthase, PRP-L: platelet rich plasma lysate, MMPs: Matrix metalloproteinases)

In SA, killing the bacteria may not be the sole option for successful disease management and strategies aimed to abrogate the progressive bone destruction by balancing the heightened immune response is equally important. Although the interplay between host cytokines, interleukins, host immune cells , bacterial clearance and progression of infection is a highly complex network and is beyond the scope of the present review, but few important therapeutic targets with possibility to use as adjunct therapy have been discussed as follows and is also depicted in Fig.4**.**

#### Matrix metalloproteinases (MMPs) based therapy

MMP’s represent important mediators in the synovial tissue destruction and pathogenesis of septic arthritis. These are calcium- and zinc dependent endopeptidases and have been classified as 24 different members (collagenases, gelatinases, stromelysins, matrilysins, membrane types etc.) [166, 167].MMP expression is elevated in septic and aseptic arthritis and this contributes to tissue destruction owing to their capability of degrading extracellular matrix (ECM) [168–171]. Bacterial products and their toxins, cytokines such as TNF-α,IL-1β and also the transcription factor, NF-kB have been implicated inactivation of these endopeptidases. As a result, inflammatory cells, synovial fibroblasts, chondrocytes, resident articular cells, synovial fibroblasts, osteoclasts start the release of MMP’s thus adding to their heightened level s[167, 172–174]. These elevated levels of MMP’s cause progressive bone and cartilage destruction in septic arthritis even after bacterial clearanc e[166, 175]. Therefore, one approach directed towards neutralization of key MMP’s and studying the effect on disease progression seems worth exploring. Gjertsson et al .[175] had highlighted the role of MMP-7 in the joint destruction in a *S. aureus* induced mouse model of arthritis. The team studied the course of infection in MMP-7 deficient mice and wild type controls which were experimentally inoculated via intravenous route with *S. aureus* LS-1 followed by development of septic arthritis. Findings showed that MMP-7 deficient mice showed reduced and less severe destruction due to arthritis both clinically and histologically although no effect on the impact on clearance of bacterial load was seen.

Recently, Sultana et al. [167] studied the protective effect of MMP-2 neutralisation on the course of *S. aureus* septic arthritis in mice and effect on cytokine regulation. MMP-2 is involved in activation of pro-IL-1β to activeIL-1β as well as in activation of other inflammatory cytokines i.e TNF-α andIL-1b [176]. Sultana and co-workers administered MMP-2 inhibitor (N-[(1, 10-Biphenyl)-4-ylsulfonyl]-D-phenylalanine) at a dose of 5 mg/kg given intraperitoneally 24 h post- infection with *S. aureus* and continued up to 15 days. Results depicted that as compared to untreated animal group, the animals treated with MMP-2 inhibitor showed a) higher reduction in associated swelling of the joints b) significantly reduced bacterial counts seen in synovial tissue at day 3 and day 9 post-infection c) significant decrease in levels of TNF-α, IL-1β , IL-6, IL-12 and d increase in IL-10 , decrease in myeloperoxidase (MPO) enzyme levels. These findings clearly indicate that neutralisation of MMP-2 represents a potential target to prevent joint destruction and regulate the cytokine levels during the arthritic episode. Further, Sultana and co-researchers [174] also evaluated the effect of combined therapy of MMP-2 and tumor necrosis factor receptor 1 antibody (TNFR1) on episodes of *S. aureus* induced septic arthritis in mice. Combined treatment group showed marked reduction in bacterial counts and low levels of pro-inflammatory cytokines in serum and synovial tissues as well as low arthritis index. Also, expression of cyclooxygenase (COX-2) and iNOS was significantly reduced in the combination group thus suggesting that such combination therapy represents a promising option in reducing the bacterial burden in the infected joint tissue as well as in decreasing the cartilage destruction and associated inflammatory damage in septic arthritis.

#### IL-33 receptor (ST2) Deficiency/Blockage as possible Target

Interleukin-33 (IL-33) is a member of the IL-1 cytokine family and it binds to its receptor ST2 expressed on activated mast cells and Th2 cells. The IL-33/ST2 axis plays key roles in joint inflammation and immune mediated diseases. Staurengo-Ferrari et al .[177] investigated the role of IL-33 receptor (ST2) deficiency on the outcome of septic arthritis. Results depicted that ST2−/− mice showed higher reduction of hyperalgesia and lower paw oedema scores. Also, wild type mice showed increased levels of TNF-α and IL-1β , higher cell infiltration as compared to the ST2−/− mice. The bacterial clearance was also higher from joint and spleen as seen in ST2-/- mice and the infection remained localized and this was possibly due to the fact that higher expression of iNOS and higher levels of nitric oxide (NO) was observed in ST2-/- mice. Thus, it was concluded that ST2 deficiency was associated with induction and enhancement of Th1 cell types leading to activation of neutrophils and macrophages with IFN-γ production, enhanced iNOS expression and improved bacterial killing resulting in effective resolution of infection thus suggesting that ST2 deficiency is beneficial in *S. aureus*-induced septic arthritis.

#### IL-15 blockage as possible target

Hennigson et al. [178] investigated the role of IL-15 in *S. aureus* mediated arthritis. They used both IL-15knockout as well as wild-type control mice treated with anti-IL15 antibodies. Systemic arthritis was induced in mice by injecting (i.v) toxic shock syndrome toxin (TSST)–1 secreting strain i.e *S. aureus* LS-1.Results showed that after inoculation, comparatively less severe arthritis was observed in mice with deleted IL-15 gene and in the mice group treated with antibody to IL-15 unlike wild-type mice. The severity of synovitis and degree of joint and cartilage destruction was significantly less with reduced number of osteoclasts seen in gene knockout mice as well as mice treated with anti-IL-15 antibody as compared with normal wild type controls. This indicates that IL-15 is a mediator of joint destruction and serves as a potential therapeutic target.

#### IL-10 Adjunct therapy

Another cytokine essential for ameliorating the course of *S. aureus* induced arthritis is IL-10.This anti-inflammatory cytokine has been shown to promote bacterial clearance and down-regulates cell mediated immune functions acting as an important immune-regulatory cytokin e[179, 180]. Study involved Balb/c mice (wild type as well as IL-10 gene deficient) that were inoculated with high dose of bacteria. IL-10 gene knockout mice developed a more severe and destructive course of infection with higher bacterial burden in associated organs i.e blood and kidneys when compared to wild type control group. This highlighted that recombinant IL-10 may have a beneficial effect on the treatment outcome and represents a possible adjunct to antibiotic treatment.

#### IL-4 Blockage as possible Target

Hultgren et al. [181] studied the role of IL-4 , a Th2 cytokine in experimentally induced *S. aureus* septic arthritis in IL-4-deficient C57BL/6 mice (IL-4−/−) and their congenic controls (IL-4+/+) challenged with TSST-1 producing strains of *S. aureus*. The IL-4−/− mice showed reduced joint inflammation and also decreased bacterial load in joints and kidneys, lesser weight loss and lower mortality score as compared to the congenic controls. This highlighted the role of IL-4 in promoting septic arthritis associated severity due to its inhibition of bacterial clearance during the *S. aureus* infection thus hinting towards use of IL-4 blockage as one of the treatment targets for ameliorating the disease pathology.

#### Platelet-rich plasma (PRP) based treatment

PRP is made by extracting donor’s whole blood which can also be pooled for use as regenerative therapy to treat and manage various musculoskeletal conditions. However, recently PRP has gained attention to exhibit potent anti-biofilm effects as well. Pooled platelet rich plasma lysate (PRP-L) has been suggested as an alternative strategy to augment the current antimicrobial treatment against infectious arthritis since *S. aureus* and *Staphylococcus epidermidis* are capable of forming floating biofilm like aggregates in both human and bovine synovial fluid. This may be a strong evading mechanism adopted by these bacteria leading to relapse of the infection despite prolonged antibiotic therapy in septic arthritis. Recently, Gilbertie et al .[182] demonstrated the potential efficacy of PRP-L formulations against synovial fluid biofilm aggregates in an in vitro equine model of infectious arthritis. For this, equine synovial fluid was collected and infected with *S. aureus*. This led to biofilm like clumps or aggregates when incubated for a two hour period in the synovial fluid. This was followed by treatment of the infected synovial fluid with PRP formulations alone as well as with aminoglycoside. Results indicated that the PRP formulations displayed significant anti-biofilm properties with marked reduction in bacterial load and displayed synergism when given along with amikacin. Further, lysis of PRP and pooling of the PRP lysate (PRP-L) also exhibited higher anti-bacterial activity against *S. aureus* which strongly advocates its further exploration as a valuable therapeutic adjunct therapy.

Box-1: Other Recent Developments against SA

**Table:** 

• Cho et al .[183] developed a novel treatment for end-stage pyogenic arthritis of the hip that consisted of developing antibiotic-loaded cement femoral head spacers. This technique was tested in 10 patients suffering from acute hip arthritis and significant joint destruction. Results demonstrated that the novel femoral head spacer technique showed promising outcomes as seen by decreased pain in the affected hip, better control of the infection with reduced burden of *S. aureus* and Streptococcus species with preserved proximal femoral bone and soft tissue tension thus overall improving the joint function and mobility in the treated patents. • Hsu et al. [184] developed a novel electrosprayed multi pharmaceutical-loaded Nanoparticle system for direct knee injections for treatment of native septic arthritis. The nanoparticles consisted of lidocaine, vancomycin, ceftazidime–eluting poly (D,L–lactide–co–glycolide) (PLGA). The biodegradable electrosprayed nano/microparticles released high concentrations of antibiotics into the synovial knee tissue of rabbits for more than 2 weeks which was well above the MIC_90_ for *S. aureus*. • Schulz et al. [185] reported the use of a novel diagnostic method termed the “Sepsis MetaScore” (SMS) which is an 11-mRNA host immune blood signature. This represents a rapid blood test that can distinguish between bacterial inflammation and non-infectious causes of inflammation thus directing correct treatment to be followed at the earliest. SMS also exhibited a higher degree of sensitivity and accuracy in diagnosing septic joints as compared to other diagnostic biomarkers (ESR, WBC and CRP) that do not help to provide information about bacterial induced or non-bacterial inflammation of the joints. • Similarly, serum Procalcitonin levels (PCT) also represent a sensitive marker for differentiating between septic arthritis and non-septic arthritis [186]. A recent meta-analysis that consisted of 10 studies including 838 patients was aimed to study the usefulness of serum procalcitonin (PCT) as a potential diagnostic marker for correct detection of early septic arthritis (SA) Zhao et al. [187]. Study results indicated and advocated that serum PCT levels represent a sensitive and specific marker with higher diagnostic value than the classical CRP based test and was also to distinguish between SA from non-SA. • Recently, Sultana and Bishayi [188] highlighted the potential use of a drug i.e etoposide that kills the monocyte/macrophage population as a useful adjunct therapy as tested in the mouse model of *S. aureus* induced SA. Mice were treated with etoposide given subcutaneously post bacterial inoculation. Results showed that the severity of arthritis was lower in the etoposide treated mice as monitored by the overall arthritis index, histopathological picture and decreased levels of pro-inflammatory cytokines, lower levels of reactive oxygen species and reduced levels of MMP-2.	

## Conclusion

*S. aureus* represents one of the problem pathogens frequently isolated from cases of septic arthritis and being associated with the highest treatment failure rates. To further worsen, there has been increased involvement of MRSA in cases of septic arthritis with higher rates of mortality than MSSA cases. Despite aggressive surgical procedure and long term antibiotic therapy, complete eradication of the pathogen may not occur leading to a chronic condition worsened with prolonged inflammation and subsequent irreversible joint issue damage. Moreover, depending upon the poor antibiotic susceptibility in case of treating resistant strains, clinical picture may turn towards need for amputation or may lead to life threatening situations such as sepsis. This calls for novel treatment modalities for acute and chronic SA effective even against the drug-resistant strains and capable of down-regulating the deranged immune responses to decrease the tissue damage. The suggested non-antibiotic approaches against SA have shown promise in various in vitro and non-human model based studies. However, what is learned from laboratory or animal models cannot be applied to humans without undergoing sufficient randomised controlled trials after ethical approval. Nonetheless, the intervention strategies discussed in the review hold string potential to augment the standard antimicrobial protocols leading to decrease in the associated morbidity and mortality rates thus enabling to advance the treatment options for septic arthritis in near future.

## Data Availability

Not applicable

## Abbreviations

MRSA: Methicillin resistant *S. aureus*
MSSA: Methicillin sensitive *S. aureus*
MMP: Matrix metalloproteinases
PCT: Pro-calcitonin
CRP: C- reactive protein
PRP: Platelet-rich plasma
CA-MRSA: Community acquired methicillin resistant *S. aureus*
HA-MRSA: Hospital acquired methicillin resistant *S. aureus*
SA: Septic arthritis
SF: Synovial fluid
TSST: Toxin shock syndrome toxin
DAIR: Debridement, antibiotics and implant retention
HBD: human β-defensin
